# *Trypanosoma cruzi* Utilizes the Host Low Density Lipoprotein Receptor in Invasion

**DOI:** 10.1371/journal.pntd.0000953

**Published:** 2011-02-01

**Authors:** Fnu Nagajyothi, Louis M. Weiss, David L. Silver, Mahalia S. Desruisseaux, Philipp E. Scherer, Joachim Herz, Herbert B. Tanowitz

**Affiliations:** 1 Department of Pathology, Albert Einstein College of Medicine, Bronx, New York, United States of America; 2 Department of Medicine, Albert Einstein College of Medicine, Bronx, New York, United States of America; 3 Department of Biochemistry, Albert Einstein College of Medicine, Bronx, New York, United States of America; 4 Internal Medicine, Touchstone Diabetes Center, University of Texas, Southwestern Medical Center at Dallas, Dallas, Texas, United States of America; 5 Biophysics and Molecular Genetics, University of Texas, Southwestern Medical Center at Dallas, Dallas, Texas, United States of America; New York University School of Medicine, United States of America

## Abstract

**Background:**

*Trypanosoma cruzi*, an intracellular protozoan parasite that infects humans and other mammalian hosts, is the etiologic agent in Chagas disease. This parasite can invade a wide variety of mammalian cells. The mechanism(s) by which *T. cruzi* invades its host cell is not completely understood. The activation of many signaling receptors during invasion has been reported; however, the exact mechanism by which parasites cross the host cell membrane barrier and trigger fusion of the parasitophorous vacuole with lysosomes is not understood.

**Methodology/Principal Findings:**

In order to explore the role of the Low Density Lipoprotein receptor (LDLr) in *T. cruzi* invasion, we evaluated LDLr parasite interactions using immunoblot and immunofluorescence (IFA) techniques. These experiments demonstrated that *T. cruzi* infection increases LDLr levels in infected host cells, inhibition or disruption of LDLr reduces parasite load in infected cells, *T. cruzi* directly binds recombinant LDLr, and LDLr-dependent *T. cruzi* invasion requires PIP2/3. qPCR analysis demonstrated a massive increase in LDLr mRNA (8000 fold) in the heart of *T. cruzi* infected mice, which is observed as early as 15 days after infection. IFA shows a co-localization of both LDL and LDLr with parasites in infected heart.

**Conclusions/Significance:**

These data highlight, for the first time, that LDLr is involved in host cell invasion by this parasite and the subsequent fusion of the parasitophorous vacuole with the host cell lysosomal compartment. The model suggested by this study unifies previous models of host cell invasion for this pathogenic protozoon. Overall, these data indicate that *T. cruzi* targets LDLr and its family members during invasion. Binding to LDL likely facilitates parasite entry into host cells. The observations in this report suggest that therapeutic strategies based on the interaction of *T. cruzi* and the LDLr pathway should be pursued as possible targets to modify the pathogenesis of disease following infection.

## Author Summary

*Trypanosoma cruzi*, an intracellular protozoan parasite that causes Chagas disease in humans and results in the development of cardiomyopathy, is a major health problem in endemic areas. This parasite can invade a wide variety of mammalian cells. The mechanisms by which these parasites invade their host cells are not completely understood. Our study highlights, for the first time, that the Low Density Lipoprotein receptor (LDLr) is important in the invasion and the subsequent fusion of the parasitophorous vacuole with host lysosomes. We demonstrate that *T. cruzi* directly binds to LDLr, and inhibition or disruption of LDLr significantly decreases parasite entry. Additionally, we have determined that this cross-linking triggers the accumulation of LDLr and phosphotidylinositol phosphates in coated pits, which initiates a signaling cascade that results in the recruitment of lysosomes, possibly via the sorting motif in the cytoplasmic tail of LDLr, to the site of adhesion/invasion. Studies of infected CD1 mice demonstrate that LDLs accumulate in infected heart and that LDLr co-localize with internalized parasites. Overall, this study demonstrates that LDLr and its family members, engaged mainly in lipoprotein transportation, are also involved in *T. cruzi* entry into host cells and this interaction likely contributes to the progression of chronic cardiomyopathy.

## Introduction

The Low-Density Lipoprotein receptor (LDLr) (UniProtKB: P01130) is a cell surface glycoprotein that plays a critical role in cholesterol homeostasis [1]. LDLr is the patriarch of an entire class of receptors called LDL receptor related proteins (LRPs) that contain similar structural modules [2]. The mature LDLr is a modular type I transmembrane protein of 839 amino acids and is composed of a number of functionally distinct domains that can function independently of each other [3], [4]. The N-terminus of the receptor contains three types of extracellular modules consisting of cysteine-rich repeats, three epidermal growth factor precursor (EGFP) regions, and O-linked oligosaccharides followed by a membrane spanning domain. The C-terminus domain of the receptor contains a signal sequence (NPXY) that is needed for receptor binding to clathrin pits and internalization [5]. The most important physiologic ligand for the receptor is Low Density Lipoprotein (LDL). Members of the LDLr superfamily bind a variety of ligands including lipoproteins, proteinases and proteinase-inhibitor complexes, and transport them into endosomes in the cell [6]. The functional properties of LDLr family members include clustering of receptors into clathrin-coated pits mediated by adaptor proteins, a pH sensitive ligand uncoupling mechanism, and recycling of the receptors back to the cell surface after dissociation of ligands. The transcription of LDL receptor is regulated by intracellular cholesterol and extracellular stimuli such as TNFα, IL-1β, TGF-β and insulin [7]–[9]. The signaling pathways leading to activation of Protein Kinase C (PKC), Protein Kinase A (PKA) and intracellular Ca^2+^ mobilization are also involved in LDLr expression [10]. LDL-containing immune complexes upregulate LDLr transcription. Interestingly, *Pseudomonas* exotoxin A and a minor group of rhinoviruses have been reported to utilize LDLr members to enter into host cells [11].

Chagas disease, caused by the obligate intracellular parasite *Trypanosoma cruzi*, affects millions of people in Mexico, Central and South America. During acute infection clinical progressions may include myocarditis and/or meningoencephalitis, although most patients are asymptomatic. Chronic manifestations include irreversible cardiomyopathy and megasyndromes [12]–[14]. Current antiparasitic treatments are not effective for chronic infection. *T. cruzi* invades a wide variety of mammalian cells including macrophages, smooth muscle cells, striated muscle cells, fibroblasts, cardiomyocytes, and adipocytes [15], [16]. In its vertebrate host this parasite is transmitted from cell to cell by non-replicating motile trypomastigotes which are capable of invading host cells; following invasion trypomastigotes transform into amastigotes which replicate intracellularly. In contrast to many intracellular pathogens that avoid contact with host cell lysosmes, *T. cruzi* requires the low pH environment of lysosomes to initiate egress from the parasitophorous vacuole and delivery to the host cell cytoplasm where replication begins [17]–[19] after approximately 24 hours post-invasion.

The molecular mechanism(s) of invasion by this parasite and the associated regulatory pathways have been the subject of intense investigation for many years. Two models of invasion, a lysosomal dependent, and a phosphotidylinositol phosphates (PIPs) pathway have been suggested for *T. cruzi* invasion. The lysosomal dependent pathway postulates that *T. cruzi* elicited signals evoke the early recruitment of host cell lysosomes to the cytosolic face of the plasma membrane at the parasite attachment site where the localized fusion of lysosomes provide membrane for the nascent parasitophorous vacuole [20]. The proposed lysosome independent PIP dependent parasite entry pathway is based on host cell PI3K signaling as a key regulator of *T. cruzi* invasion [20], [21]. However, neither of these models explains the precise mechanism(s) by which this parasite traverses the host cell permeability barrier and interacts with the lysosomal compartment.

Recent studies have demonstrated that *T. cruzi* activates many cell membrane receptors such as Toll-like receptors (TLRs), kinins (B1/B2 sub types), receptor tyrosine kinases, TGF and EGF receptors and that the activity of these receptors is required for optimal parasite binding and/or invasion [22]–[25]. In addition, invasion results in the activation of ERK/MAPK signaling pathways. Taken together these studies reveal that *T. cruzi* activates many signaling pathways involving diverse receptors on the host cell surface in preparation for internalization.

Our study highlights, for the first time, that LDLr is involved in the trafficking of lysosomes to the parasitophorous vacuole containing trypomastigotes and that inhibition or disruption of LDLr affects the intracellular parasite load. We also report that LDLr expression is upregulated in infected mouse hearts and LDL/LDLr is associated with the amastigotes (pseudocysts) in the heart tissue of infected mice. The accumulation of LDL and LDLr in the heart probably contributes to the pathogenesis of chagasic heart disease. The LDL/LDLr pathway could represent a new therapeutic target for modulating *T. cruzi* infection.

## Materials and Methods

### Parasitology and pathology

The Brazil strain of *T. cruzi* was maintained in C3H/He mice (Jackson Laboratories, ME). Six to 8 week old male CD-1 mice were obtained from Charles River Laboratories (Wilmington, MA) and infected IP with 5×10^4^ trypomastigotes. The serum and heart tissues were collected at 15, 20 and 30 days p.i. The mice were anesthetized with isoflurane and about 75 µl of blood is collected from the orbital venous sinus. The mice were then observed for recovery from anesthesia and returned to their cages. The parasitemia was determined using a Neubauer hemocytometer. Hearts were fixed in 10% buffered formalin and paraffin sections were stained with IFA. The animal experiments were approved by the Institutional Animal Care and Use 200Committees (IACUC) of Albert Einstein College of Medicine (No.20100204). Parasites were also maintained in L_6_E_9_ myoblasts as previously described [26].

### Mammalian cell culture

Human Foreskin Fibroblast (HFF) (ATCC CRL 1475), rat cardiomyocyte H9c2 and 3T3-L1 (ATCC CL 173) cell lines are maintained in our laboratory using standard methods as previously published [27], [28].

### Materials

All the cell culture reagents used in these experiments were obtained from Cellgro (Mediatech Inc.), primary antibodies were obtained from Abcam (MA) and secondary fluorescence antibodies were obtained from Invitrogen (CA) unless other suppliers are specifically mentioned in the text. For each experiment a minimum of 4 mice were used per group and each experiment has been repeated thrice.

### Immunoblot analysis

Cell lysates were prepared as previously described [29]. An aliquot of each sample (40 µg protein) was subjected to 7.5% SDS-PAGE and the proteins were transferred to nitrocellulose filters for immunoblot analysis. LDLr specific rabbit monoclonal antibodies (1∶2000 dilution, ab52818 Abcam, MA) and horseradish peroxidase- conjugated goat anti-rabbit immunoglobulin (1∶5000 dilution, Amersham Biosciences) were used to detect specific protein bands (explained in Figure Legends) using a chemiluminescence system [29]. GAPDH (1∶5000 dilution, mouse monoclonal Ab8245, MA and secondary antibody horseradish peroxidase conjugated goat anti-rabbit 1∶2000 dilution, Amersham Biosciences) was used to normalize protein loading.

### Immunofluorescence analysis (IFA)

Fibroblasts were cultured on cover slips to 80% confluence and then infected with trypomastigotes (3.1×10^6^/cm^2^ surface area of culture plates) for 10, 20 and 30 minutes. The fibroblast cultures were fixed with 4% paraformaldehyde, permeabilized with 0.1% Triton ×100 (30min) and stained for LDLr/clathrin/PIP/LAMP2/LDL using specific primary antibodies rabbit monoclonal to LDLr (Ab52818), mouse monoclonal to Clathrin (1∶200, Ab2731), mouse monoclonal to PIP2 (1∶300, Ab11039), rat monoclonal LAMP1 (1∶150, Hybridoma bank, 1D4B), rat monoclonal to LAMP2 (1∶150, Hybridoma bank, ABL93) and goat anti human LDL (1∶10, Sigma L 8016) respectively used with the concentrations as recommended by the manufacturers and Alexa fluor 594 (goat anti rabbit or anti mouse IgG 1∶500 dilution; Invitrogen, CA), or Alexa 488 (goat anti rabbit or anti mouse or anti rat IgG 1∶500 dilution; Invitrogen CA). The cells were stained with DAPI (blue) to detect nuclei following manufacturer's protocols (www.abcam.com/technical). Images were obtained and analyzed by fluorescence microscopy using an inverted Olympus IX71 with a HQ2 CCD camera and a Nikon Microphot-FXA with Spot camera software. IFA of paraffin embedded tissues were performed as previously published [30].

### Inhibition of *T. cruzi* invasion by PCSK9

HFF cells were pretreated with recombinant proprotein convertase subtilisin/kexin type 9 (0.5ug of PCSK9/cm^2^ surface area) for 1h at 37°C. PCSK9 treated and untreated cells were incubated with trypomastigotes (3.1×10^6^/cm^2^ surface area) for 1h at 37°C. The cells were washed (5 times in phosphate buffered saline (PBS, 7.2), fixed with 4% paraformaldehyde, permeabilized with 0.1% Triton X-100 (30 min incubation) and stained with DAPI. The number of parasites/2000 host cells was counted under the microscope (40×). The total number of parasites counted in PCSK9 untreated cells was considered as 100%.

### Double staining IFA of infected cells to differentiate bound and invaded parasites

Fibroblast cells were incubated with parasites (MOI 5∶1) for 1h, washed to remove unbound parasites, fed with fresh medium and incubated at 37°C. At 4, 15 and 24h post infection, the cells were fixed with 4% paraformaldehye, blocked in 3%BSA, incubated with anti-parasite mouse serum (serum of infected CD1 mice 1∶20 dilution) and secondary antibody fluorescent Alexa 480 (green) to stain parasites bound to the cell surface. Then the cells were permeabilized with 0.2% Triton X-100, blocked in 3%BSA, incubated with anti- parasite mouse serum and secondary antibody fluorescence Alexa 594 (red) to stain intracellular parasites.

### Binding of parasite with recombinant human LDLr

Trypomastigotes (1.8×10^6^) were washed twice in PBS and incubated with 5 µg of recombinant hu-LDLr (2148-LD/CF, R&D Systems, Inc.) for 1h at room temperature (final concentration 10ng/ul). The incubation mixture was centrifuged at 5000 rpm for 5min, washed twice in DMEM (Dulbecco's Modified Eagle Medium) and surfaced on lysine treated cover slips for 20 min before fixing with 4% paraformaldehyde (30min). IFA was performed for bound parasites using LDLr specific monoclonal antibody (1∶100 dilution, for 1h at 37°C) and IgG goat Alexa fluor 594 (1∶500 dilution for 1h at 37°C).

As an alternative method we used recombinant hu-LDLr dye conjugate (Alexa fluor 488, prepared as per Invitrogen protocol) to incubate with trypomastigotes for 1hr at room temperature. The parasites were then centrifuged at 5000 rpm for 5 min, washed with DMEM (2 times) and surfaced on lysine treated cover slips for 20 min before fixing with 4% paraformaldehyde. GAPDH dye conjugate was prepared as above and used as control.

### RNA extraction and qPCR analysis

Total RNA was extracted from the heart tissue of CD1 infected mice using Trizol reagent (Invitrogen). Further cleaning up of RNA was performed using RNeasy minikit (QIAGEN Sciences, Maryland) according to the manufacturer's instructions. Reverse transcription of total RNA and the quantitative PCR was carried out as described earlier using iQ5 BioRad system [16]. The LDLr mRNA levels were detected using PCR arrays designed by SABiosciences (PAMM-030) following manufacturer's instructions.

### DNA extraction and qPCR analysis of parasite load

Wild type and LDLr KO cells (mouse embryonic fibroblast) [31] were incubated with trypomastigotes (3.1×10^6^/cm^2^ surface area) for 1h at 37°C. The cells were washed four times in PBS to remove unbound parasites and incubated in DMEM containing 10% FBS for 68 hrs at 37C. Parasite load in these cells was quantitated by real-time PCR as previously described [29].

### Quantification of serum LDL, HDL and triglycerides in CD1 mice

Serum collected at day 15, 20 and 30 p.i. from CD1 infected and uninfected mice were used to quantitate serum LDL, HDL and triglyceride levels using E2HL-100 (EnzyChrom AF HDL and LDL/VLDL Assay Kit) following manufacturer's protocol.

## Results

### *T. cruzi* infection increases LDLr levels in infected host cells

**Figure 1:**
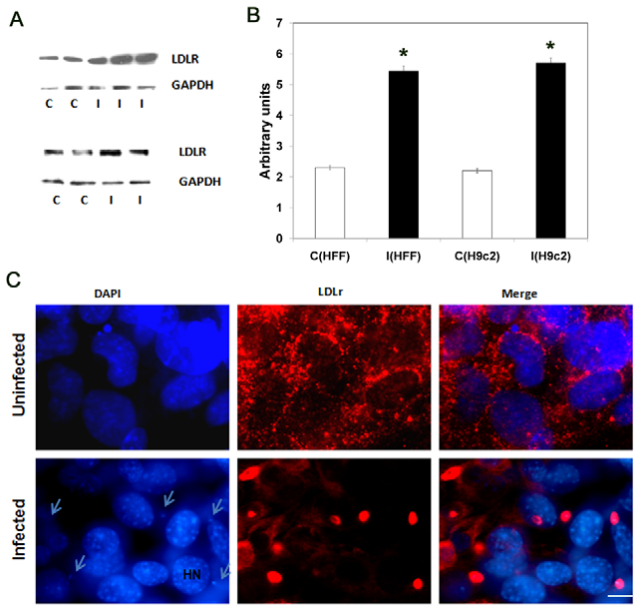
LDLr expression in *T. cruzi* infected host cells. A. Immunoblot analysis of LDLr in infected cells. Increased LDLr levels were observed in both infected HFF cells (upper panel) and H9c2 cell lines (lower panel) after 1h incubation with parasites. B. Quantitative analysis of immunoblots. Arbitrary units of the expressed LDLr proteins normalized to respective GAPDH levels represented in the bar histogram as quantitated using Alpha Ease FC software. C- control, I- infected, C(HFF)- control HFF cells, I(HFF)- infected HFF cells, C(H9c2)- control H9c2 cells and I(H9c2)- infected H9c2 cells (n = 4 and p<0.05 represented as star). C. Distribution of LDLr in uninfected and infected fibroblast cells. IFA demonstrated an even distribution of LDLr at cell membrane in uninfected cells, but clustering of LDLr in infected cells after 15 minutes of incubation with parasites (arrows). (Host nucleus≈HN, bar represents 50µm).

Endocytosis of LDL receptor (LDLr) in association with calcium mobilization, its subsequent trafficking to lysosomes, and the release of ligands at low pH are processes reminiscent of those involved in *T. cruzi* invasion. Interestingly, some rhinoviruses use LDLr members to enter into host cells [11]. We hypothesized that *T. cruzi* may utilize host LDLr to enter the host cells. The association of LDLr with *T. cruzi* invasion and infection was therefore investigated using *in vitro* infection of human fibroblast cells (HFF) and murine cardiomyocytes (H9c2). Immunoblot analysis of LDLr protein levels in cell lysates from infected HFF and H9c2 cells using LDLr specific monoclonal antibodies demonstrated that HFF and H9c2 cells incubated with *T. cruzi* had a two-fold increase in LDLr protein levels within 1h post-infection (p.i.) (Figure 1A and B). The monoclonal LDLr antibodies used in these studies were raised against the synthetic peptide corresponding to residues from the C-terminus of the human LDLr. To determine the distribution of LDLr proteins in uninfected and infected cells immunofluorescence analysis (IFA) was performed using LDLr specific monoclonal antibodies (Figure 1C). IFA of uninfected fibroblasts demonstrated an even distribution of LDLr around the cell membrane. In contrast, there was a clustering of LDLr in infected cells (10 min p.i.) at the cell membrane suggesting a role for LDLr in infection. These antibodies did not cross react with *T. cruzi* alone (Figure S1).

### Inhibition or disruption of LDLr reduces parasite load in infected cells

**Figure 2:**
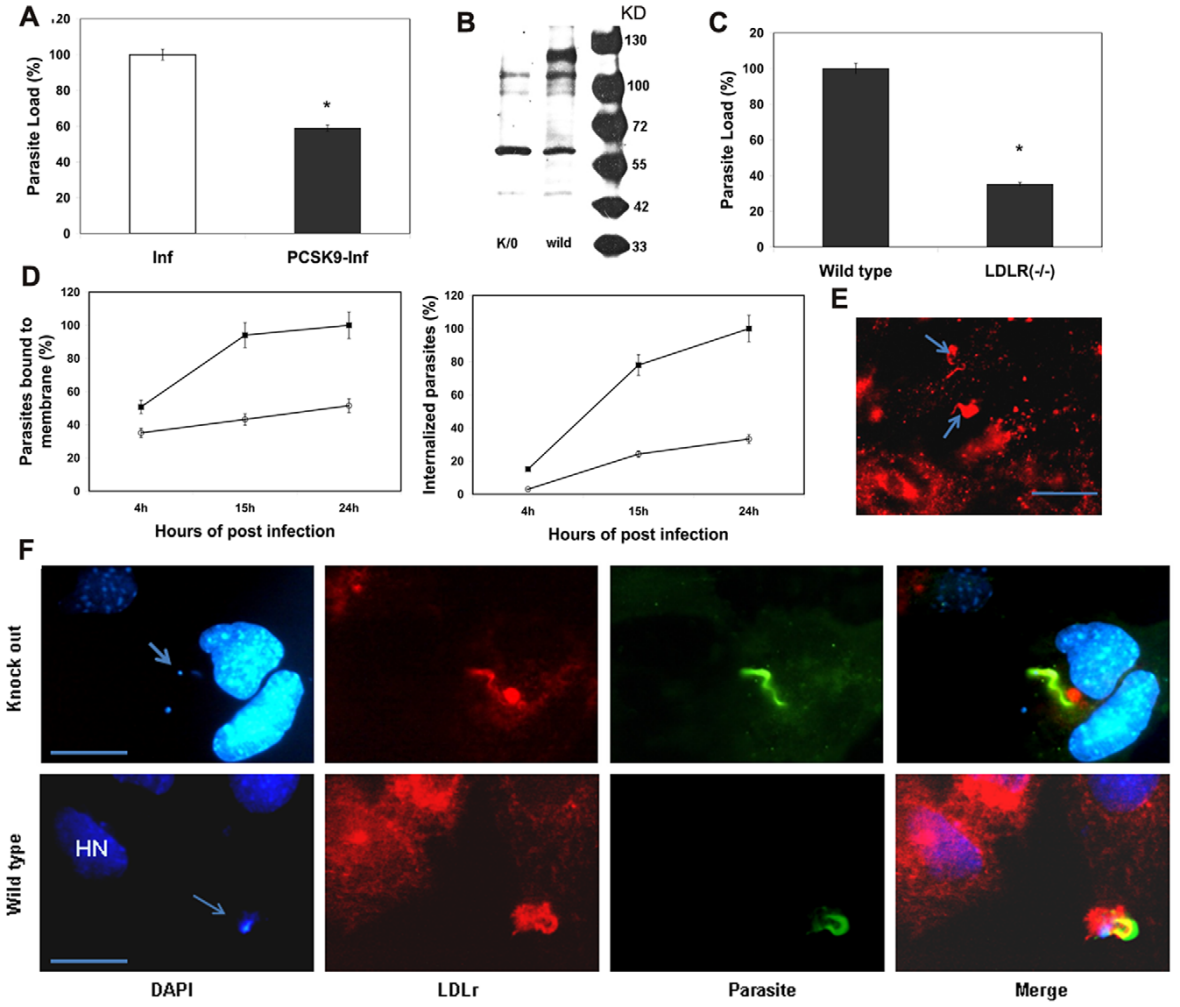
Inhibition/disruption of LDLr reduces parasite load in infected cells. A. PCSK9 pretreatment reduced parasite invasion. Incubation of parasites with PCSK9 pretreated HFF cells reduced parasite load by degrading LDLr. The bar histogram demonstrates the parasite load in PCSK9 pretreated and untreated HFF cells (n = 4 and p<0.05 represented as an “*”). B. Immunoblot analysis of LDLr in wild type and LDLr KO cells. Immunoblot analysis using monoclonal LDLr antibodies confirmed the lack of full length LDLr at 120 KD (represented by arrow) in KO cells. However, LDLr KO cells express a truncated LDLr which lacks the LDL binding domain (27). C. Quantitative analysis of parasite load in infected wild type and LDLr KO cells. Reduced parasite load in LDLr KO cells after 68h of infection compared to infected wild type cells demonstrated by real time PCR analysis. Bar histogram represents the parasite load (%) in infected cells. D. Kinetic studies of parasite binding and invasion in LDLr KO cells. Lack of full length LDLr protein in LDLr KO cells reduced the number of parasites attached to cell membrane (Upper line) and also retarded the parasite invasion (lower line), demonstrated by IFA as described under experimental procedures. E. IFA of LDLr in infected LDLr KO cells. IFA using monoclonal LDLr antibodies (red) demonstrated the clustering of disrupted LDLr around the structures similar to parasites (arrow) in KO cells (bar represents 50µm). F. Co-localization of parasites with disrupted LDLr in infected KO and wild type cells. Double staining IFA using infected mouse serum (green) and monoclonal LDLr antibodies (red) demonstrated the co-localization of parasites with disrupted LDLr (top panel) and full length LDLr (lower panel). (bar represents 50µm).

To investigate the role of LDLr in parasite trafficking, we pre-incubated HFF cells with exogenous recombinant proprotein convertase subtilisin/kexin type 9 (PCSK9), an enzyme which binds to the extracellular domain of LDLr and induces LDLr degradation [32]. The PCSK9 pretreated cells demonstrated a 42% reduction in *T. cruzi* invasion compared to untreated cells (Figure 2A). Immunoblot analysis of PCSK9 pretreated cells demonstrated no significant difference in LDLr levels between uninfected and infected HFF cells (Figure S2).

To determine whether parasites can invade the cells in the absence of LDLr, we infected embryonic fibroblast cells derived from LDLr KO mice [31]. Immunoblot analysis confirmed the absence of full length LDLr (120 kDa) protein in KO cells compared to wild type cells (Figure 2B). It should be noted, however, that these LDLr-KO cells express a truncated LDLr as previously demonstrated by Ishibashi [31]. This truncated version (based on the original gene KO) lacks the domains for binding LDL and for internalization. When cells were incubated with *T. cruzi* for 1h and assayed at 68 hrs there was a 62% reduction in total parasite load in LDLr-KO cells compared to wild type using a qPCR method (Figure 2C). Parasites bound strongly to the cell surface in these LDLr KO cells and could not be dislodged even with extensive washings with PBS. A kinetic study of parasite invasion using a double staining IFA method demonstrated that parasites rapidly invaded wild type cells, but were retarded (70%) from invading the LDLr-KO cells (Figure 2D). Binding versus internalization assays performed after 15 hrs of incubation with parasites using double staining IFA showed significantly less internalized parasites in KO cells compared to wild type (Figure S3). We observed a reduced LDLr protein (truncated) expression in KO cells compared to wild type full length LDLr (data not shown). IFA demonstrated that clustering of the disrupted LDLr still occurred at the cell membrane and that the disrupted LDLr remained associated with internalized parasites in infected KO cells similar to that of wild type (Figure 2E). Parasite binding was noted to occur in the vicinity of this truncated LDLr in these KO cells (Figure 2F). We do not know whether this association of truncated protein with the parasite is involved in parasite internalization (30% compared to wild type) or if other members of LDLr family are involved in the absence of full length LDLr. Overall, these results supported the hypothesis that LDLr plays an important role in mediating *T. cruzi* invasion.

### *T. cruzi* exploits LDLr to invade mammalian cell

**Figure 3:**
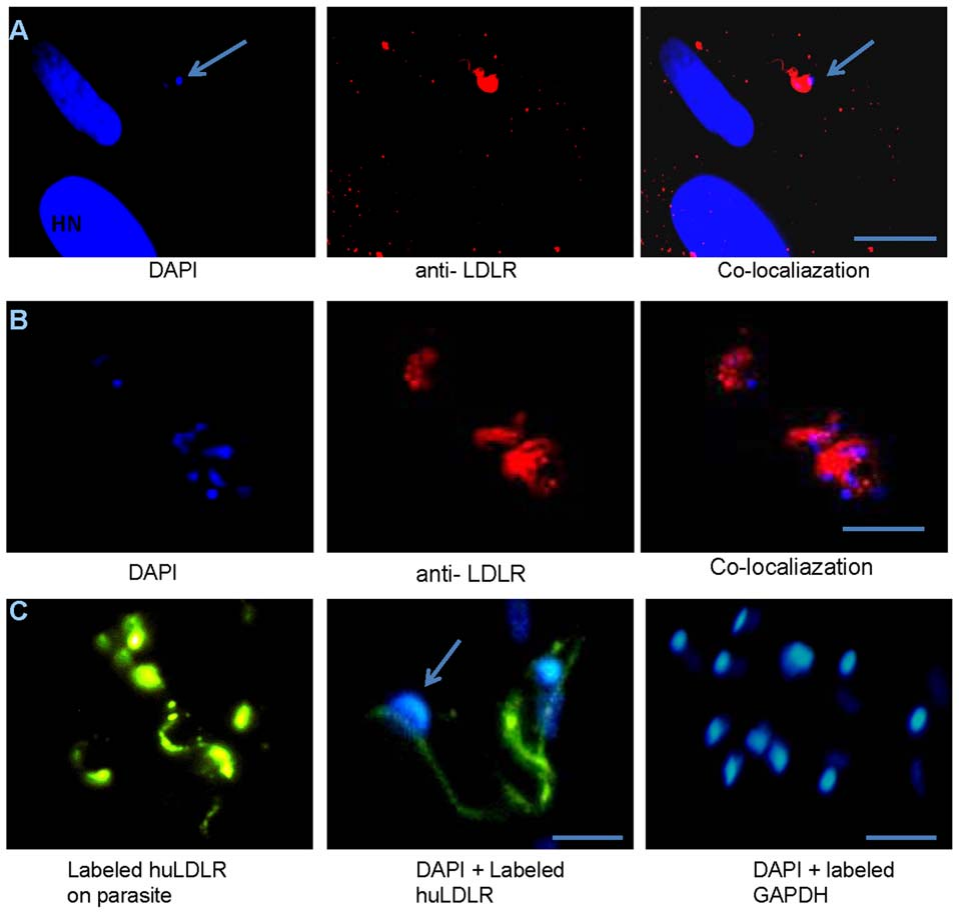
*T. cruzi* targets LDLr during invasion as demonstrated by IFA. A. Association of LDLr with *T. cruzi* during invasion. IFA of LDLr in Infected HFF cells showed the co-localization of LDLr (red) with parasite. The cells were stained with DAPI (blue) to detect nucleus (bar represents 50µm). B. Binding of parasite to recombinant human LDLr. IFA demonstrated the direct binding of parasite with exogenously added recombinant huLDLr using monoclonal antibodies specific for LDLr (bar represents 50µm). C. Direct binding of parasite to Fluorescence labeled recombinant huLDLr. Fluorescent labeled huLDLr bound to the parasites on their cell surface demonstrated by fluorescent microscopy (left) and stained with DAPI (center). Alexa fluor 488 labeled GAPDH dye conjugate is used as a control (right). (bar represents 10µm).

The activation of various cell surface receptors and signaling pathways by *T. cruzi* has been reported by other investigators [22]–[25]. To examine if trypomastigotes utilize LDLr to activate certain signaling pathways to enter the host cells or if LDLr-assisted pathways are involved in internalization, we performed IFA to observe the localization of LDLr in infected cells. IFA of HFF cells incubated with trypomastigotes demonstrated an association of LDLr with internalized parasites within 10 min of incubation (Figure 3A).

It was previously reported that *T. cruzi* has an affinity for binding to LDL [33]. We performed IFA of thoroughly washed parasites using LDL specific antibodies and observed no signals for the presence of LDL (data not shown). To ascertain whether trypomastigotes can directly bind to LDLr or use LDL as a bridge to bind to LDLr, we performed binding studies using recombinant human LDLr (huLDLR Ala22-Arg788). We incubated washed parasites with recombinant LDLr and carried out IFA as described in experimental procedures. IFA using monoclonal LDLr antibodies demonstrated the association of recombinant LDLr with the parasites (Figure 3B). An alternative experiment with fluorescent labeled LDLr also confirmed direct binding between parasites and the LDLr (Figure 3C). Fluorescent labeled GAPDH (control protein) did not bind to parasites and the monoclonal LDLr antibody did not bind to the parasite in the absence of preincubation of parasites with LDLr.

### LDLr- Dependent *T. cruzi* invasion requires PIP2/3

**Figure 4:**
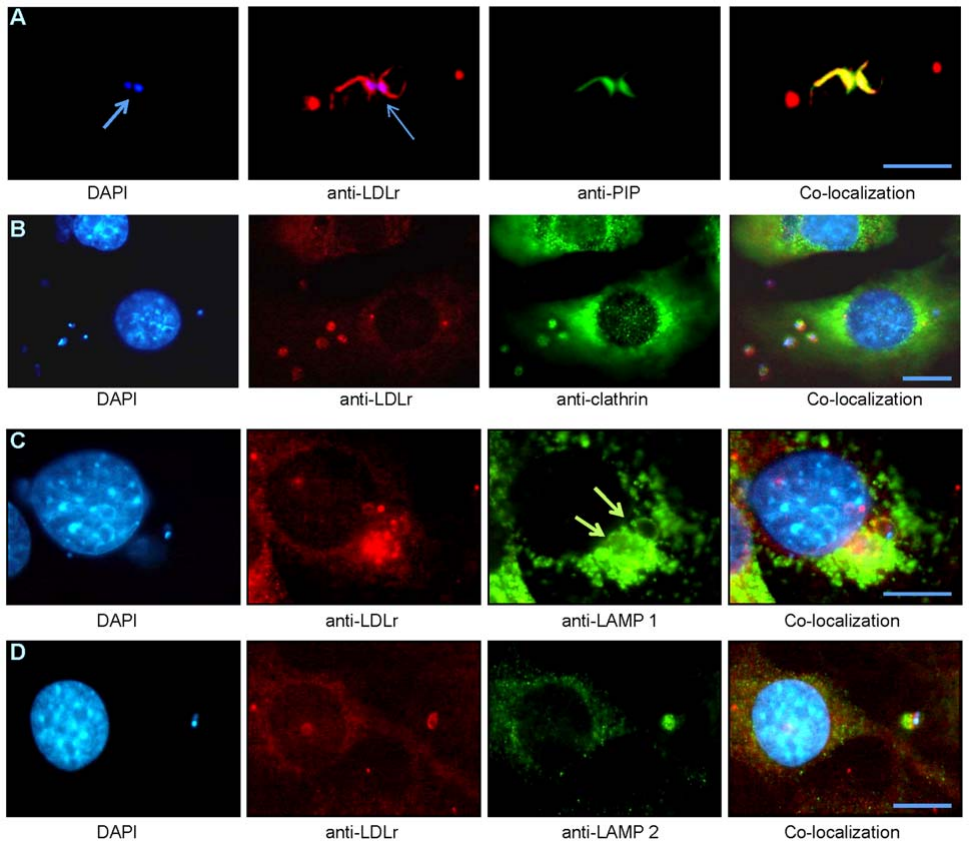
Role of LDLr and its associates in trafficking *T. cruzi* into host lysosomes. A. PIP2/3 is associated with LDLr during invasion. Co-localization of PIP2/PIP3 (green) and LDLr (red) with parasite (DAPI) is demonstrated by triple staining IFA. B. Parasite utilizes LDLr/clathrin complex to enter host cells. Triple staining IFA of infected 3T3-L1 cells demonstrated the co-localization of clathrin (green) with LDLr (red) and parasites (DAPI). C. Presence of LDLr during lysosomal fusion with parasites during infection. Parasite trafficking to lysosomes (LAMP1- green) by LDLr (red) is demonstrated by the co-localization lysosome, LDLr and parasite as shown by IFA. (green arrow represents the presence of LAMP1 around parasitophorous vacuoles. D. Co-localization of total LAMP-2 with parasites during infection. Triple staining IFA demonstrated the co-localization of LAMP-2 (green) with LDLr (red) and parasites (DAPI) (bar represents 50µm).

The endocytosis of LDLr and its associated adaptor proteins, including clathrin and PIPs, has been extensively studied. Accumulation of PIP2 and PIP3 at the penetration site of trypomastigotes has been reported [34]. To further confirm the association of PIPs to LDLr during parasite invasion we performed double staining IFA with antibodies specific for LDLr and PIP2/3. IFA demonstrated the co-localization of LDLr and PIPs with invaded parasites in infected HFF cells (Figure 4A).

It had been previously reported that inhibition of dynamin (a protein involved in clathrin-mediated endocytosis), drastically diminished *T. cruzi* entry in both phagocytic and non-phagocytic cells [35]. To investigate the involvement of clathrin in *T. cruzi* invasion, we performed IFA of 30 min infected HFF cells using clathrin specific monoclonal antibodies. The results as demonstrated in Figure 4B corroborate the involvement of clathrin in the invasion of extracellular parasites into host cells. These antibodies did not cross react with *T. cruzi* alone (Figure S1).

### *T. cruzi* targets LDLr in trafficking to host cell lysosomes

The functional properties of LDLr family members include clustering of receptors into clathrin-coated pits mediated by adaptor proteins, a pH sensitive ligand uncoupling mechanism, and recycling of the receptors back to the cell surface after dissociation of ligand in endosomes [1]. We wanted to ascertain whether parasites dissociate from LDLr as soon as they enter host cells or co-exist while fused with endosomes/lysosomes in order to gain entrance to the acidic environment important for transformation from trypomastigotes to amastigotes. Therefore, we employed double staining IFA using lysosome associated membrane proteins (LAMP-1 and 2) specific antibodies (Figure 4C and 4D). Fibroblast cells infected with trypomastigotes for 30 min showed the presence of lysosomes surrounding the invaded parasites in association with LDLr. These antibodies did not cross react with *T. cruzi* alone (Figure S1).

### Role of LDLr in *in vivo T. cruzi* infection

**Figure 5:**
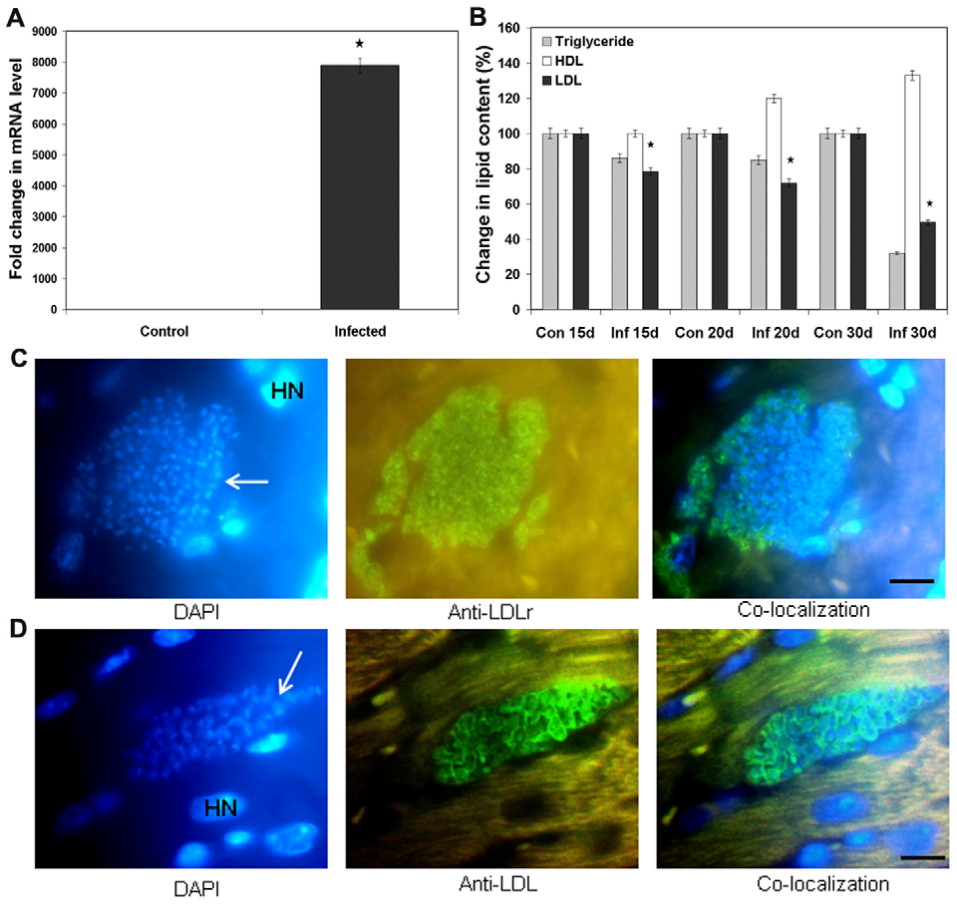
Role of LDL/LDLr in acute stage of *T. cruzi* infected CD1 mice. A. Increased mRNA levels of LDLr in heart. qPCR analysis demonstrated a massive increase in LDLr mRNA levels in 15dpi CD1 mouse heart. B. Serum lipid profile of infected mice. A significant reduction in LDL and total triglycerides in the serum of infected mice is detected using colorimetric assay. C. Association of LDLr with parasite nests in infected heart. IFA of hearts demonstrated the co-localization of LDLr (green) with parasites (pseudocysts) in infected (15dpi) CD1 mice (bar represents 10µm). D. Accumulation of LDL with parasites in infected mouse heart. IFA showed the presence of LDL (green) co-localized with parasites (bar represents 10µm).

To investigate the role of LDLr in a *T. cruzi* infected mouse model, we infected CD1 mice with trypomastigotes and analyzed for LDLr mRNA levels in heart tissue 15 day p.i. qPCR demonstrated a significant increase in the mRNA levels of LDLr up to 8000 fold in infected heart tissue compared to control mice (Figure 5A). A serum lipid analysis was performed to examine any changes in LDL, HDL and triglyceride levels in infected mice compared to uninfected mice (Figure 5B). LDL levels in infected mice significantly decreased with time (22% by 15 day p.i. and 50.5% by 30 day p.i.) compared to control mice. IFA of paraffin embedded heart tissue of infected mice (15 day p.i.) using LDLr specific monoclonal antibodies (Figure 5C) demonstrated the co-localization of LDLr to the specific area surrounding invaded parasites in heart tissue. We also performed IFA of LDL using LDL specific polyclonal antibodies in these tissues and the results confirmed an accumulation of LDL along with LDLr around intracellular parasites in the hearts of infected mice (Figure 5D). These results demonstrate that in addition to the increase in LDLr mRNA levels in infected tissues there was also a localization of LDLr as well as LDL to areas of pseudocysts containing thousands of parasites.

## Discussion

LDLr family members share similar structural homology and are involved in lipoprotein and other ligand endocytosis events. Internalization of ligands by LDLr is a complex process which requires a vast assembly of structural coat components and a host of accessory proteins to drive the endocytic machinery. Many signaling pathways and secondary messengers are involved in this process. The mechanisms involved in LDLr endocytosis are similar to that of *T. cruzi* internalization such as, calcium mobilization, fusion with endosomes/lysosomes, and the requirement of an acidic pH environment. We therefore explored the possible involvement of LDLr in *T. cruzi* invasion.

The *in vitro* and *in vivo* observations in the current manuscript confirm that *T. cruzi* utilizes the LDLr in their host cell invasion process. The interaction between *T. cruzi* and host cells has been extensively reviewed [36]–[38]. Earlier reports demonstrate that a variety of host receptors become activated during *T. cruzi* binding and invasion. For example, activation of TLR2 mediated Rab-5 in *T. cruzi* invasion has been explored. TNF-α, interleukins and cytokines are regulated by TLR-2 activation. Two other receptors namely, “transforming growth factor β receptor and bradykinin receptor” have also been reported to be involved in *T. cruzi* infection [23], [25]. Melo-jorge and Pereira Perrin demonstrated the involvement of receptor tyrosine kinases during *T. cruzi* invasion [24]. Based on these reports, we propose that *T. cruzi* binds to host cell membrane receptors and activates many signaling pathways mainly involved in cell proliferation, PI3kinase activation, MAPK signaling and transcription factors, since all these components are known to positively regulate LDLr transcription [7]–[10].

Our results demonstrate that the parasites directly bind to LDLr and inhibition or disruption of LDLr resulted in a reduced rate of invasion. This mechanism of invasion is associated with PIPs and clathrin. It had been previously reported that inhibition of dynamin (a protein associated in clathrin-mediated endocytosis), drastically diminished *T. cruzi* entry in both phagocytic and non-phagocytic cells [35]. Earlier reports have demonstrated the accumulation of PIP2/3 around the parasite penetration site and parasitophorous vacuoles and that inhibition of PI3 kinase resulted in decreased parasite entry [21], [34], [39]. Here, we report that phosphotidylinositol bis phosphate (PIP2) co-localized to the LDLr/parasite complex (Figure 4A). These data are consistent with the earlier observation of a lysosome independent pathway for parasite invasion [17]–[20]. It is probable that *T. cruzi* uses the sorting motif in the cytoplasmic tail of LDLr to recruit the host lysosomes to the site of invasion, which provides the acidic environment to the parasite for its transformation to amastigotes. IFA demonstrated the co-localization of lysosomes around the parasite associated LDLr complex (Figure 4C). While these results are consistent with a role for LDLr in *T. cruzi* internalization and trafficking to host lysosomes, the exact mechanism through which LDLr recruits the lysosomes to the site of invasion will require further studies. Overall, these observations indicate that both of the current models that exist for *T. cruzi* invasion (i.e lysosome-dependent and PIPs dependent) are part of the same model in which the LDLr complex machinery connects and completes the process of invasion by this pathogenic microbe.

Our studies employing wild type and LDLr KO cells suggest that the presence of full-length LDLr facilitates the binding and internalization of parasites. Disruption of LDL binding domains retarded both parasite binding and invasion. IFA revealed that parasites could still associate with the truncated LDLr expressed in LDLr-KO cells. The KO lacks the LDL binding domain but contains other functional regions of the LDLr including the C terminus. The monoclonal LDLr antibodies employed were raised against the synthetic peptide corresponding to residues from the C-terminus of the human LDLr. The NPXY motif at the C-terminal sequence of LDLr is involved in the internalization signaling [5] and persists in the KO construct. Further investigations will be necessary to determine if other members of the LDLr family are also involved in parasite invasion in the absence of full length LDLr or if the truncated LDLr itself was involved in the reduced rate (30% of wild type) of parasite internalization seen in the LDLr KO cells.

Acutely infected mice displayed a significant decrease in plasma LDL levels. In addition, LDL was increased at areas where parasites were present in the heart. The infection-associated increase in phospholipids, triglycerides, and fatty acids could contribute to the pathogenesis of chagasic heart disease [40]. Our data strongly suggest that LDLr and its family members play an important role in *T. cruzi* invasion and the subsequent lysosomal recruitment that facilitates transformation of trypomastigotes into amastigotes. LDL may facilitate parasite entry and also contribute to LDL-parasite immune complexes regulating LDLr levels [41]. Further research on the mechanism by which this parasite interacts with the host LDLr/clathrin complex is justified. In addition, the observations in this report suggest that therapeutic strategies based on the interaction of *T. cruzi* and the LDLr pathway should be pursued as possible targets to modulate the consequences of infection.

## Supporting Information

Figure S1 LDLr, Clathrin, LAMP antibodies do not cross react with parasite alone. Double staining IFA demonstrated no cross reactivity with parasites alone. Equivalent amounts of LAMP-1 and LAMP-2 antibodies were used and demonstrate that none of these antibodies cross reacts with *T. cruzi* (bar represents 50 µm).
(1.03 MB TIF)

Figure S2 Immunoblot analysis of LDLr in PCSK 9 treated cells. Cell lysates from *T. cruzi* infected (1h p.i.) PCSK9 treated cells were analyzed for LDLr expression by immunoblot. No change in LDLr level was observed between uninfected and infected cells in contrast to PCSK9 untreated cells (Figure 1A).
(0.08 MB TIF)

Figure S3 Binding versus internalization in LDLr KO cells. Double staining IFA of wild type (A) and LDLr KO (B) cells demonstrated the reduced number of internalized parasites (bright red) in KO cells compared to wild type and the presence of bound parasites in both wild type and KO cells (yellow) (bar represents 50 µm).
(0.83 MB TIF)
