# Supplementary figures and images for: Trypanosoma cruzi Utilizes the Host Low Density Lipoprotein Receptor in Invasion
Source: PLoS Negl Trop Dis. 2011 Feb 1;5(2):e953. doi: 10.1371/journal.pntd.0000953 (PMC3051337; doi:10.1371/journal.pntd.0000953)

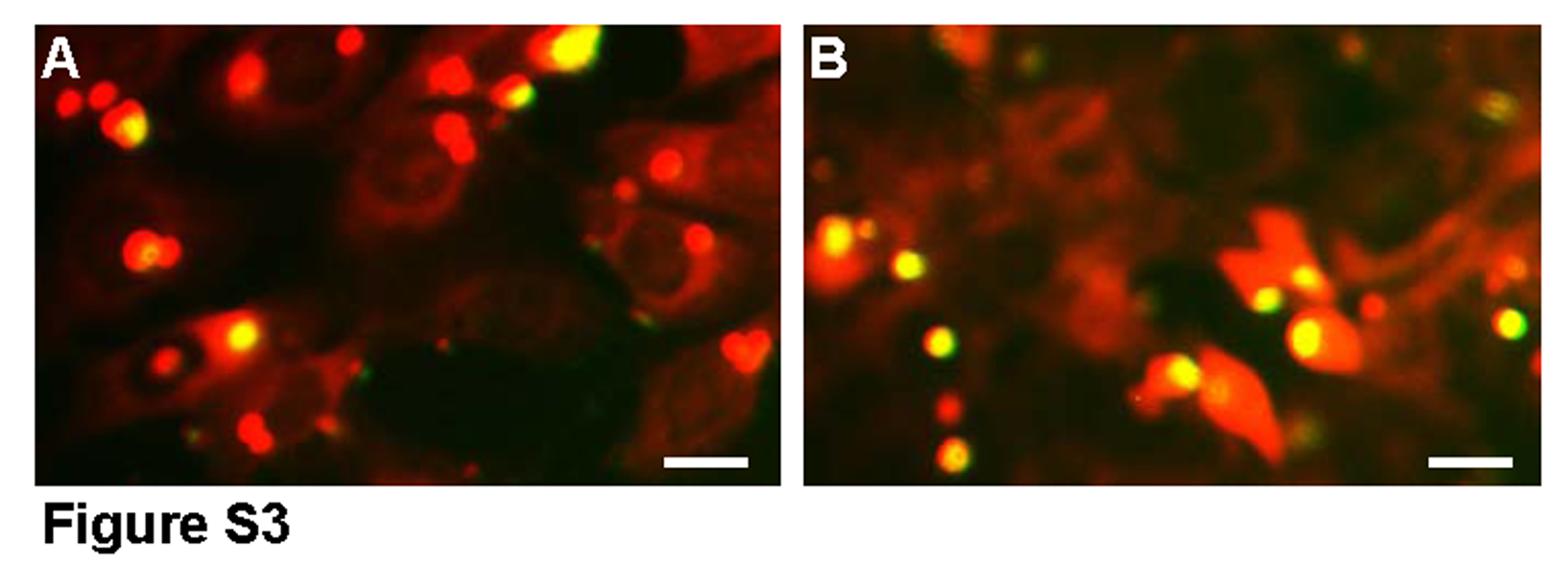

Supplement: Figure S1 — LDLr, Clathrin, LAMP antibodies do not cross react with parasite alone. Double staining IFA demonstrated no cross reactivity with parasites alone. Equivalent amounts of LAMP-1 and LAMP-2 antibodies were used and demonstrate that none of these antibodies cross reacts with T. cruzi (bar represents 50 µm). (1.03 MB TIF) [file pntd.0000953.s001.tif]

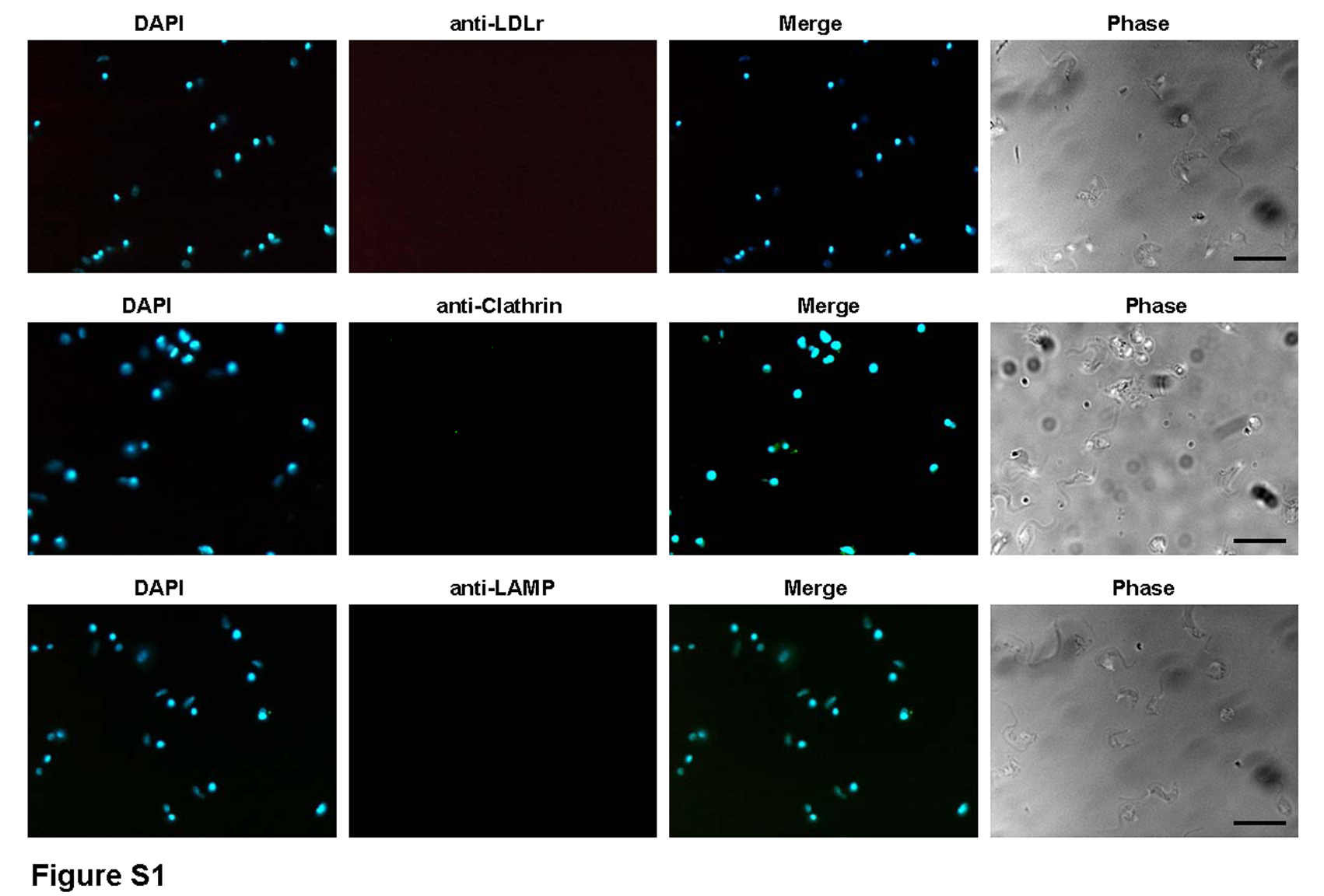

Supplement: Figure S2 — Immunoblot analysis of LDLr in PCSK 9 treated cells. Cell lysates from T. cruzi infected (1h p.i.) PCSK9 treated cells were analyzed for LDLr expression by immunoblot. No change in LDLr level was observed between uninfected and infected cells in contrast to PCSK9 untreated cells (Figure 1A). (0.08 MB TIF) [file pntd.0000953.s002.tif]

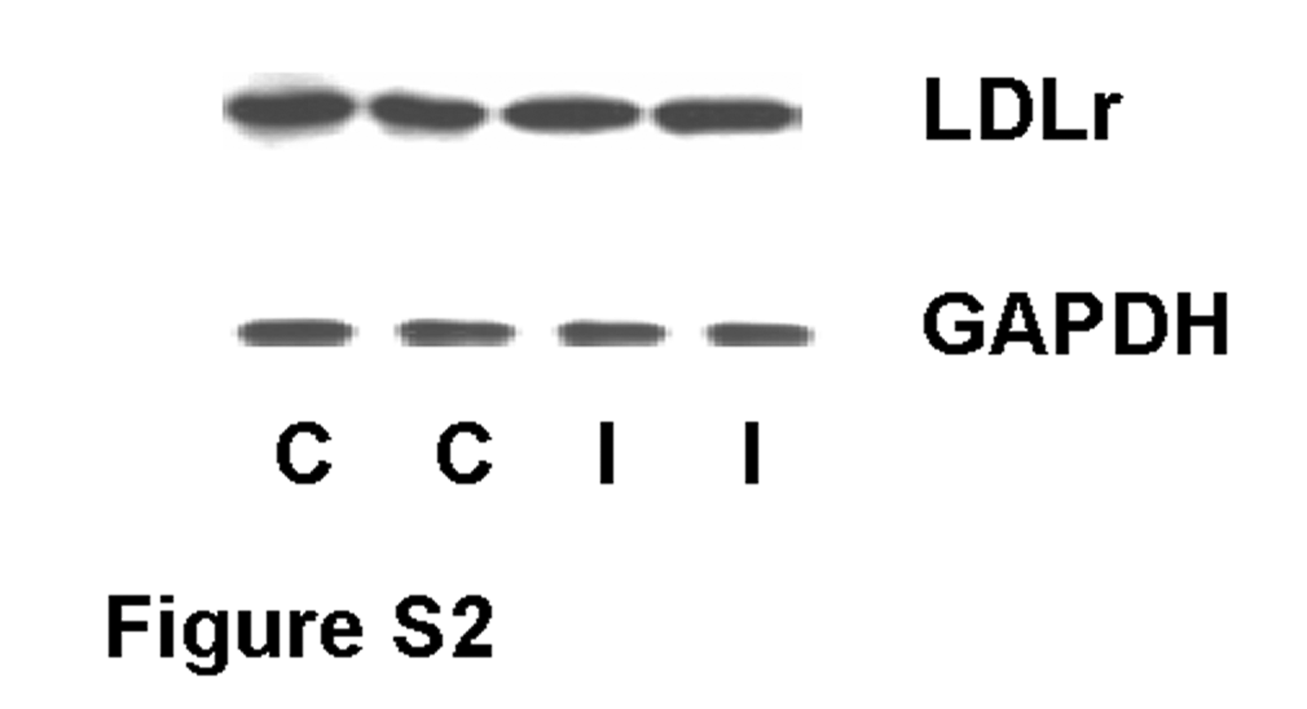

Supplement: Figure S3 — Binding versus internalization in LDLr KO cells. Double staining IFA of wild type (A) and LDLr KO (B) cells demonstrated the reduced number of internalized parasites (bright red) in KO cells compared to wild type and the presence of bound parasites in both wild type and KO cells (yellow) (bar represents 50 µm). (0.83 MB TIF) [file pntd.0000953.s003.tif]
